# Gaucher disease carrier with gestational thrombocytopenia and anemia: a case report

**DOI:** 10.1186/s13256-022-03388-6

**Published:** 2022-05-13

**Authors:** Takako Sugiura, Arisa Fujiwara, Takasugi Yo, Kana Kashinoura, Chihiro Hayase, Yumiko Taura, Yasuhiro Kawarabayashi, Yasuyuki Hasuo, Shinji Ogawa

**Affiliations:** Department of Obstetrics, Perinatal Center, National Hospital Organization Kyushu Medical Center, 1-8-1, Jigyohama Chuo-ku, Fukuoka, 810-8563 Japan

**Keywords:** Gaucher disease, Gestational thrombocytopenia, Idiopathic thrombocytopenic purpura

## Abstract

**Background:**

Gaucher disease is an autosomal recessive inborn error of metabolism that causes disorders of blood, bone, and central nervous system as well as hepatosplenomegaly. We present the case of a carrier of Gaucher disease with gestational thrombocytopenia and anemia that required blood transfusion therapy.

**Case presentation:**

A 24-year-old Nepalese primipara was diagnosed with idiopathic thrombocytopenia at 12 weeks of gestation. Her platelet count had reduced to 30,000/µL at 21 weeks of gestation, and the hemoglobin content reduced to 7.6 g/dL at 27 weeks of gestation. As she did not respond to any medication, blood transfusion was performed. A female infant weighing 2677 g was delivered vaginally at 39 weeks of gestation. On the 78th day of puerperium, the platelet count of the mother recovered to 101,000/µL, and the hemoglobin content recovered to 12.5 g/dL. The infant had convulsions, respiratory depression, wheezing, systemic purpura, and exfoliation of the epidermis at birth. The infant was diagnosed with Gaucher disease at 37 days of age and passed away at 82 days of age. Subsequently, the parents were diagnosed as carriers of Gaucher disease.

**Conclusion:**

As carriers of this disease do not usually show symptoms, it is imperative to provide information regarding disease management for future pregnancies.

## Introduction

Thrombocytopenia during pregnancy is observed in approximately 10% of all pregnancies. Gestational thrombocytopenia is quite common, in which the platelet count of the mother is usually around 70,000–100,000/µL [1, 2]. However, the platelet count returns to normal range within 1–2 months after childbirth. In our case, the mother was diagnosed with idiopathic thrombocytopenic purpura (ITP) and anemia during pregnancy. She underwent blood transfusion as other therapies turned out to be ineffective. After birth, the infant was diagnosed with Gaucher disease, an autosomal recessive disorder, and the mother was diagnosed as a disease carrier. Gaucher disease is characterized by thrombocytopenia, anemia, hepatosplenomegaly, and bone disorders. However, disease carriers are usually asymptomatic; therefore, the clinical course of the disease might be different between disease carriers and disease-affected patients. In this report, we present the case of a carrier of Gaucher disease with gestational thrombocytopenia and anemia.

## Case presentation

**Fig. 1:**
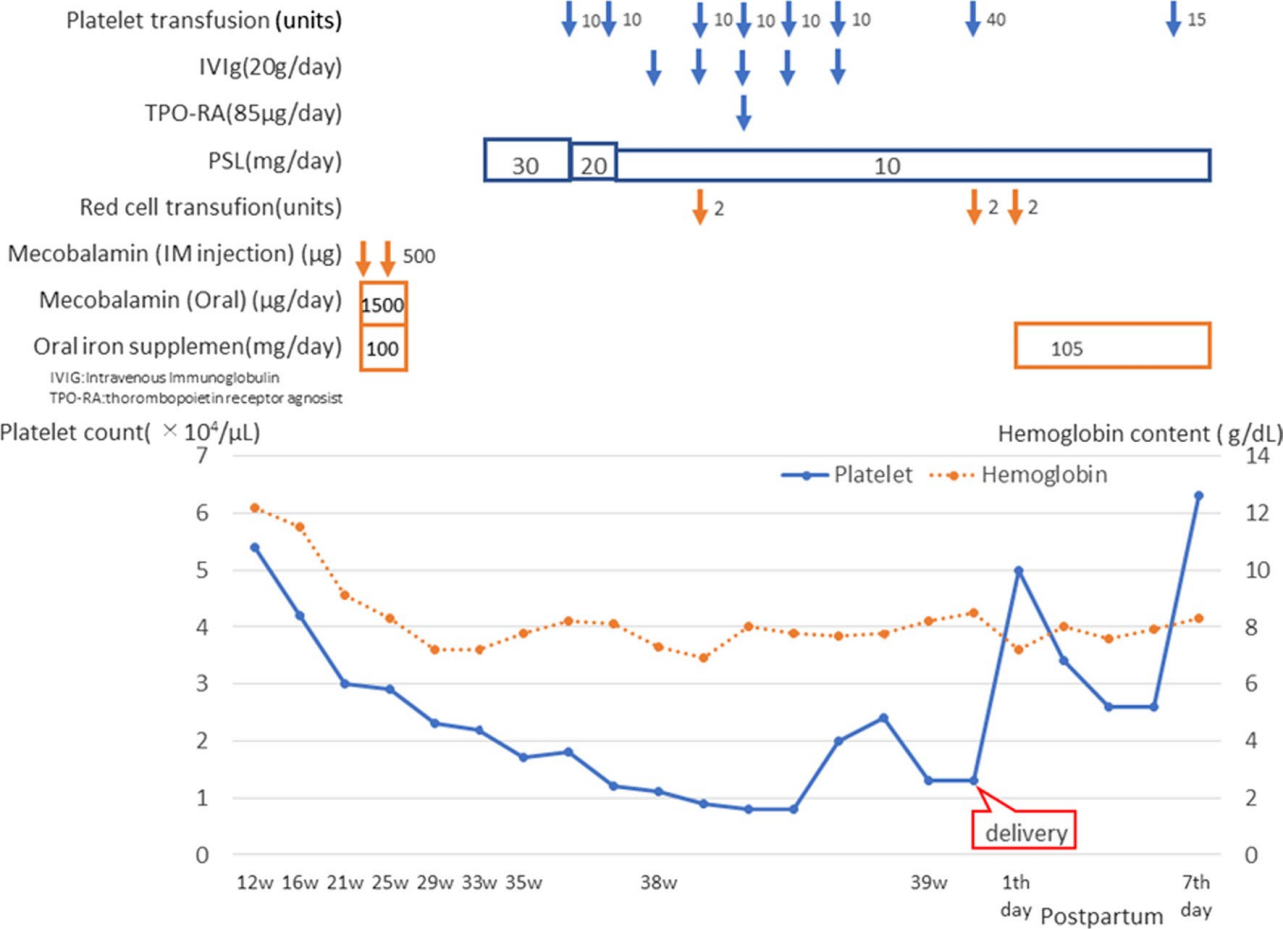
Progress of platelet count and hemoglobin content under treatment details. She developed severe thrombocytopenia and anemia during pregnancy. All medications were ineffective, and she required blood transfusion therapy

A 24-year-old Nepalese primigravida with no remarkable personal or family medical history was admitted to our hospital with a platelet count of 97,000/µL at 12 weeks of gestation. Blood biochemistry analysis revealed elevated levels of platelet associated (PA) immunoglobulin G (IgG) at 214 ng/10^7^ cells, while *Helicobacter pylori* IgG, human immunodeficiency virus (HIV) antibody, hepatitis C virus (HCV) antibody, and hepatitis B surface antigen were all negative. There were no findings suggestive of systemic lupus erythematosus, antiphospholipid antibody syndrome, viral infection, or liver dysfunction. The patient was diagnosed with idiopathic thrombocytopenic purpura (ITP), and her platelet levels were monitored periodically until the end of the second trimester to assess if the platelet count will fall below 30,000/µL. At 21 weeks of gestation, her platelet count decreased to 30,000/µL. Additionally, her hemoglobin levels reduced from 12.2 g/dL at 12 weeks to 8.1 g/dL at 23 weeks and eventually to 7.6 g/dL at 27 weeks gestation. Such a decline in hemoglobin was concluded as macrocytic anemia due to a decrease in her vitamin B12 levels; however, oral administration of ferrous citrate and vitamin supplementation were ineffective. No significant abnormalities in the bone marrow were detected through bone marrow puncture. Oral administration of vitamins for anemia was stopped at 31 weeks of gestation. We started administering prednisolone, oral corticosteroid, for thrombocytopenia at 33 weeks of gestation; however, no significant change in platelet count was observed. Therefore, we started blood transfusion therapy, while continuing with 10 mg prednisolone. At the beginning of the 36th and 37th week of pregnancy, 10 units of platelets were transfused each time, which led to an increase in the platelet count from 18,000 to 40,000 and 12,000 to 27,000/µL, respectively. From the beginning of the 38th week of pregnancy, we administered high-dose immunoglobulin therapy for 5 days; however, the platelet count did not increase enough to support childbirth. Next, we administered romiplostim, a thrombopoietin receptor agonist, which had no effect on the platelet count. At a platelet count of 9000/µL, a third round of 10 units of platelet transfusion was performed, but it was ineffective in increasing the platelet count. At this stage, the anti-human leukocyte antigen (anti-HLA) antibody test was positive. To treat the anemia, 2 units of red blood cell fluids were administered, which increased the hemoglobin levels from 6.9 g/dL to 8.0 g/dL. Labor was induced in the patient on the first day of the 39th week of pregnancy. As the platelet count was only 41,000/µL, before complete dilation of the uterine ostium, 10 units of HLA-matched platelets and 20 units of concentrated platelets were transfused. A female infant weighing 2677 g was delivered vaginally. During the episiotomy suture, another 10 units of platelets were transfused. No hematoma was observed in the birth canal; however, the patient lost almost a liter of blood due to uterine atony. After delivery, the patient’s hemoglobin level was 6.5 g/dL; hence, 4 units of red blood cell fluids were transfused, and dry iron sulfate was administered orally. On the fifth day of puerperium, 15 units of HLA-matched platelets were transfused, as the platelet count was only 26,000/µL. On the sixth day of puerperium, no significant changes were observed through bone marrow puncture and spinal magnetic resonance imaging (MRI). On the seventh day of puerperium, the platelet count and hemoglobin level recovered to 63,000/µL and 8.3 g/dL respectively, and the patient was discharged (Fig. 1). All oral medications were discontinued on the 29th day of puerperium, when the platelet count was 44,000/µL and the hemoglobin level reached 10.2 g/dL. The patient was diagnosed with gestational thrombocytopenia on the 78th day of puerperium, since the platelet count spontaneously recovered to 101,000/µL. Additionally, mild splenomegaly was observed during the puerperium through transabdominal ultrasonography.

**Fig. 2:**
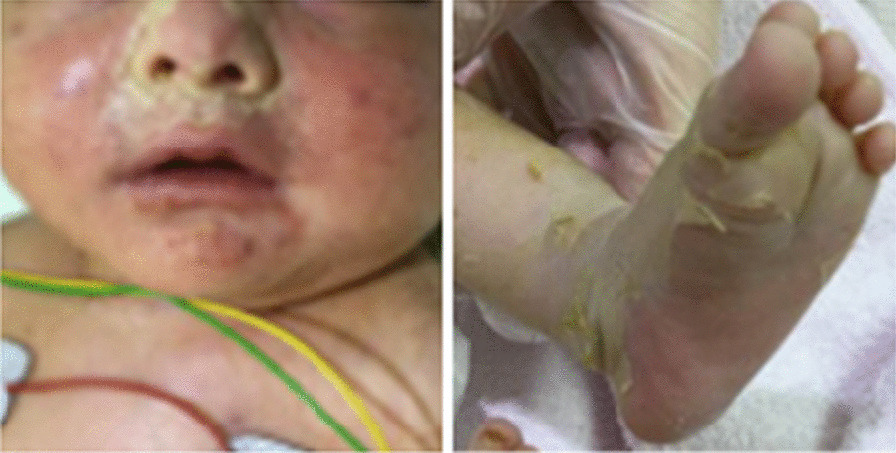
Collodion baby. The infant’s epidermal detachment of the face and limbs was remarkable. This skin finding is characteristic of perinatal Gaucher disease type 2 and is called collodion baby

The infant's Apgar score was 5 points after 1 min and 7 points after 5 minutes. She had weak breathing and started convulsions 3 minutes after birth. Some other symptoms in the infant included attenuated light reflex, midline fixation of the eyeballs, respiratory depression, and wheezing. Hypertonia and purpura were observed throughout the body, and the epidermal detachment of the face and limbs was remarkable (Fig. 2). Additionally, her platelet count was 17,000/µL, and acute subdural hematoma and hepatosplenomegaly were observed. These symptoms were refractory to therapeutic interventions such as concentrated platelets and immunoglobulins. The infant was diagnosed with Gaucher disease at 37 days of age by mass screening, and a homozygous mutation (p.L483R) was found in the glucocerebrosidase (*GBA*) gene. After receiving enzyme replacement therapy at 64 and 78 days of age, she died of suffocation at 82 days of age. The β-glucosidase activity of the patient and her husband was normal, and a heterozygous mutation in the *GBA* gene was found in both. Therefore, we diagnosed them as carriers of Gaucher disease and provided genetic counseling for future pregnancies including prenatal diagnosis such as amniocentesis and chorionic villus sampling.

## Discussion and conclusions

The causes of thrombocytopenia during pregnancy include gestational thrombocytopenia (74%), preeclampsia (21%), ITP (4.1%), and some other diseases accounting for less than 1% of the total cases [1]. Gestational thrombocytopenia is diagnosed if the platelet count recovers after delivery. During pregnancy, the symptoms of gestational thrombocytopenia and ITP are indistinguishable, and both diseases are diagnosed by exclusion [2]. In gestational thrombocytopenia, the platelet count is less than 100,000/µL in 1.0%, and less than 80,000/µL in about 0.1% of cases [3]. It is recommended to manage thrombocytopenia as ITP, in cases with platelet counts of 100,000/µL or less early in pregnancy, with declining platelet counts as gestation progresses [2]. Prednisolone therapy at 0.5–1 mg/kg/day is the first choice for ITP, and high-dose immunoglobulin therapy at 0.4 g/kg/day is administered before invasive procedures [2]. The platelet count increases to 30,000/µL in about 80%, and 100,000/µL in approximately 50% of cases administered prednisolone therapy [4]. In patients administered high-dose immunoglobulin therapy, the platelet count increases to 100,000/µL in 64% of the cases [5]. Our patient was diagnosed with ITP, and as both prednisolone and immunoglobin therapy were ineffective, a thrombopoietin receptor agonist, rarely used in pregnant cases, was administered. Despite the 80% success rate of the thrombopoietin receptor agonist [6], it was found to be ineffective. None of the therapies including transfusion could rescue the platelet count, which fell to 8000/µL at 38 weeks of gestation. In total, we administered 50 units of platelets during pregnancy and 65 units of platelets during labor and puerperium (including 25 units of HLA-matched platelets), which had no significant effect in restoring platelet levels. However, the patient’s platelet count and hemoglobin level returned to normal range spontaneously during puerperium, due to which she was diagnosed with gestational thrombocytopenia. We realized that the course of progression of gestational thrombocytopenia was strange, as platelet levels fell to 10,000/µL (extremely rare) and anemia did not respond to medication during pregnancy.

Eventually, our patient was diagnosed as a carrier of Gaucher disease, an autosomal recessive inborn error of metabolism caused by genetic mutation in the lysosomal enzyme GBA [7]. More than 300 mutations have been reported in the *GBA* gene, which is located on chromosome 1 (q21–q31) in humans. Gaucher disease affects approximately 1 in 40,000 to 1 in 60,000 newborns. A decrease in the β-glucocerebrosidase enzyme (encoded by *GBA*) activity leads to progressive accumulation of glucosylceramide in reticuloendothelial cells such as macrophages. As a result, hematological abnormalities such as thrombocytopenia and anemia, hepatosplenomegaly, and bone disorders such as bone pain and pathological fractures are observed. Glucosyl sphingosine, another substrate of β-glucocerebrosidase, accumulates in the brain and causes central nervous system disorders including respiratory dysfunction, dysphagia, eye movement disorders, convulsive seizures, intellectual regression, joint contraction, involuntary movements, and dysarthria. According to the severity of symptoms, the central nervous system disorders are classified as type 1 (non-nerve type), type 2 (acute nerve type), and type 3 (subacute nerve type). Type 1 accounts for 90–95% of Gaucher disease cases, and the age of onset for more than half of the cases is before 20 years. Splenomegaly is present in more than 90% of cases, as well as bone and nonserious hematological abnormalities. Type 2 has a classic type and a perinatal type, accounting for less than 5% and less than 1% of all Gaucher disease cases, respectively. The classic type develops around 3–6 months after birth, causes central nervous system disorders, with life expectancy being around 2 years. The perinatal type is the most severe and may lead to hydrops fetalis and intrauterine fetal death. In addition to hepatosplenomegaly and thrombocytopenia, infants affected with the perinatal type are born with ichthyosis-like skin desquamation and are called collodion babies. Central nervous system disorders develop immediately after birth in infants affected with the perinatal type of Gaucher disease, and their life expectancy is usually less than 3 months. Type 3 accounts for approximately 5% of the disease, develops by around 2 years of age, and presents with ocular motility disorder, cerebellar ataxia, epilepsy, and valvular heart disease [7, 8]. In our case, the infant exhibited central nervous system disorders immediately after birth; thus, she was diagnosed with type 2 (perinatal type) Gaucher disease.

Thrombocytopenia and anemia during pregnancy and puerperal splenomegaly are classic symptoms of Gaucher disease; however, Gaucher disease carriers are usually asymptomatic. To confirm this further, we searched for medical literature using search terms such as “Gaucher disease carrier,” “thrombocytopenia,” and “anemia” on PubMed and the Japan Medical Abstracts Society, but could not find any reports describing disease carriers who showed symptoms. In our case as well, the patient had not exhibited any symptoms such as anemia, thrombocytopenia, or bone disorders in the past. Additionally, none of her family members showed any symptoms similar to Gaucher disease.

Sickle cell disease, which has the same autosomal recessive inheritance as Gaucher disease, is symptomatic in carriers under certain conditions such as excessive exercise [9]. Since Gaucher disease is rare, few carriers have been identified. This is the first case report of a Gaucher disease carrier with blood disorder. Hence, further investigations are needed to establish management outcomes. In our case, all the medications were ineffective, and the patient recovered spontaneously during puerperium. After the newborn was diagnosed with Gaucher disease type 2, she was identified as a carrier of Gaucher disease with a heterozygous mutation in the *GBA* gene. As described in this case, Gaucher disease carriers may present with symptoms.

Gaucher disease carriers are usually asymptomatic, but in our case, hematological abnormalities such as thrombocytopenia and anemia during pregnancy and puerperal splenomegaly were observed. In the future, it will be recommended to provide Gaucher disease carriers with information about both fetal and maternal complications and management.

## Data Availability

The datasets used and/or analyzed during the current study are available from the corresponding author on reasonable request.

## Abbreviations

ITP: Idiopathic thrombocytopenic purpura
HIV: Human immunodeficiency virus
HCV: Hepatitis C virus
HLA: Human leukocyte antigen
